# Extracorporeal membrane oxygenation during pregnancy and peripartal. An international retrospective multicenter study

**DOI:** 10.1177/02676591221090668

**Published:** 2022-05-13

**Authors:** S Fill Malfertheiner, D Brodie, A Burrell, FS Taccone, LM Broman, K Shekar, C L Agerstrand, A L Serra, J Fraser, MV Malfertheiner

**Affiliations:** 1 Department of Obstetrics and Gynecology, Hospital St. Hedwig of the Order of St. John, Regensburg University, Regensburg, Germany; 2 Division of Pulmonary, Allergy, and Critical Care Medicine, 12294 Columbia University College of Physicians and Surgeons, NewYork-Presbyterian Hospital, New York; 3 Center for Acute Respiratory Failure, NewYork-Presbyterian Hospital, New York; 4 Department of Intensive Care, The Alfred Hospital, Melbourne, Australia; 5 Department of Intensive Care, Hôpital Erasme, Université Libre de Bruxelles, ULB, Brussels, Belgium; 6 ECMO Centre Karolinska, Department of Pediatric Perioperative Medicine and Intensive Care, Karolinska University Hospital, Department of Physiology and Pharmacology, Karolinska Institutet, Stockholm, Sweden; 7 Critical Care Research Group, 67567 The Prince Charles Hospital, Brisbane, Australia; 8 Department of Internal Medicine II, Cardiology and Pneumology, University Hospital Regensburg, Regensburg, Germany

**Keywords:** extracorporeal membrane oxygenation, pregnancy, peripartum, hemorrhage, influenza, pneumonia, acute respiratory distress syndrome, pulmonary embolism, amniotic fluid embolism, cardiomyopathy

## Abstract

**Introduction:**

Extracorporeal Membrane Oxygenation (ECMO) may be used in the setting of pregnancy or the peripartal period, however its utility has not been well-characterized. This study aims to give an overview on the prevalence of peripartel ECMO cases and further assess the indications and outcomes of ECMO in this setting across multiple centers and countries.

**Methods:**

A retrospective, multicenter, international cohort study of pregnant and peripartum ECMO cases was performed. Data were collected from six ECMO centers across three continents over a 10-year period.

**Results:**

A total of 60 pregnany/peripartal ECMO cases have been identified. Most frequent indications are acute respiratory distress syndrome (*n* = 30) and pulmonary embolism (*n* = 5). Veno-venous ECMO mode was applied more often (77%). ECMO treatment during pregnancy was performed in 17 cases. Maternal and fetal survival was high with 87% (*n* = 52), respectively 73% (*n* = 44).

**Conclusions:**

Various emergency scenarios during pregnancy and at time of delivery may require ECMO treatment. Peripartal mortality in a well-resourced setting is rare, however emergencies in the labor room occur and knowledge of available rescue therapy is essential to improve outcome. Obstetricians and obstetric anesthesiologists should be aware of the availability of ECMO resource at their hospital or region to ensure immediate contact when needed.

## Introduction

In case of respiratory and/or circulatory failure extracorporeal life support (ECLS) provides the opportunity to restore gas exchange and circulation in pulmonary and/or cardiac failure.^1^ Within the field of ECLS, techniques are distinguished with veno-venous Extracorporeal membrane oxygenation (VV ECMO) for respiratory and veno-arterial (VA) ECMO for circulatory support.^2^ In VV ECMO mode venous blood is removed from the patient, is pumped through a membrane oxygenator (artificial lung) and returned to the patients venous circulation. Within the membrane oxygenator carbon dioxide is removed and oxygen is added to the circulated blood. This technique provides extracorporeal gas exchange. In VA ECMO mode the device and setup is the same, only the blood is returned to the patient through an arterial cannula, this provides circulatory support in addition to the gas exchange.

ECMO therapy is an emerging therapy in critical care medicine with not only improving technology but also expanding indications.^3^ One indication with rapidly growing case numbers for example is Extracorporeal Cardiopulmonary Resuscitation (ECPR).^4^ ECPR can be defined as the implantation of a VA ECMO in a patient who experienced a sudden and unexpected cardiac arrest.^5^ In certain centers with specialized teams, it can be applied in in-hospital and even in out-of hospital cardiac arrest.^6^

While ECMO therapy is more and more established in dedicated centers worldwide, it is still rather fameless in obstetric cases. A limited number of case reports with successful applications in pregnant women or peripartal emergency scenarios exist in the literature. Ong and colleagues^7^ published a systematic review covering all ECMO cases in pregnancy or the peripartal period published between 1972 and 2017. This review showed a survival of the mother around 91% and a fetal survival of 78% . A second systematic review on all ECMO cases during pregnancy, reported acute respiratory distress syndrome (ARDS) due to H1*N*1 to be the most frequent reason for ECMO support and a maternal survival rate of 78% .^8^

A need for optimization in emergency management in this field is important, as the death of a pregnant woman or a young mother is always a tragic event. Prevention of maternal death is a key point for all health care systems.

In the United States about 700 women die each year from complications related to pregnancy. Maternal mortality review committees consider about 60% of these deaths to be preventable.^9^ With rising maternal age and an increase of obesity in pregnancy, both associated with higher risk of adverse events pregnancy-related mortality is rising.^10^

Leading causes of death included cardiovascular conditions, infection, and hemorrhage. And limited experience with obstetric emergencies was considered a contributing factor of preventable deaths.^9^ Therefore, adequate emergency and intensive care resources play a crucial role and ECLS treatment should be considered when available.

The aim of this study was therefore to give a representative picture of actual indications and pregnancy related cases in a number of international high-volume ECMO centers.

## Methods

**Figure 1.**
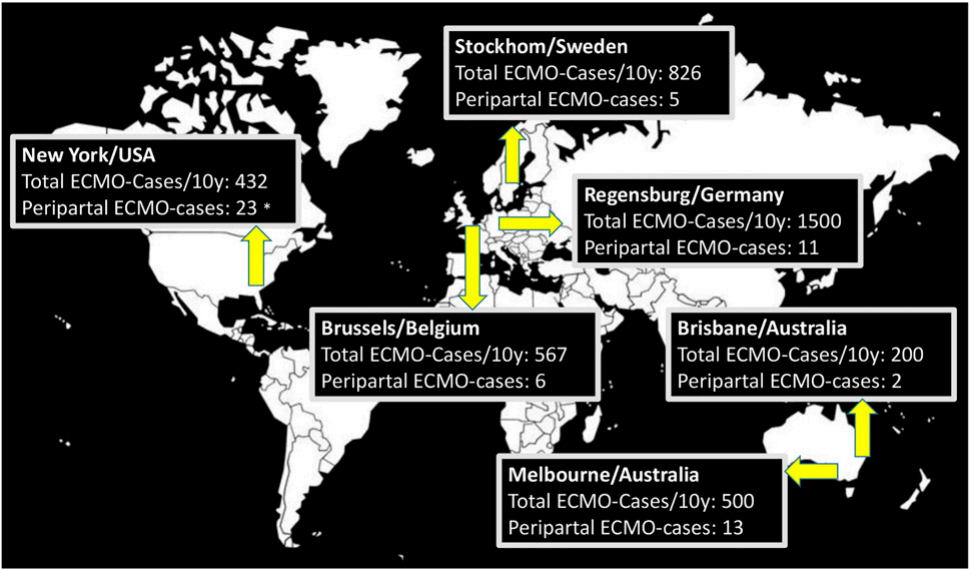
World map showing participating centers and indicating total ECMO (extracorporeal membrane oxygenation) cases over a time span of 10 years and the number of pregnancy or peripartal related ECMO cases (*12 cases are published previously).

An international retrospective, multi-center study on pregnancy-related ECMO cases was performed. Participating centers were the University Hospital Regensburg, Regensburg (Germany), Université Libre de Bruxelles, Brussels (Belgium), The Prince Charles Hospital, Brisbane (Australia), The Alfred hospital, Melbourne (Australia), Karolinska University Hospital, Stockholm (Sweden) and Columbia University, New York (USA) (Figure 1). Institutional ethics committee approval was obtained as required by local ethics committees.

Institutional retrospective ECMO data bases were screened to identify all ECMO cases related to pregnancy or the peripartal period. Defined as “peripartal period” were pregnancy itself and up to 2 months postpartum. Included in the analysis have been all pregnancy/peripartal-related cases receiving either VV or VA ECMO between January 2009 and December 2018. No exclusion criteria were applied.

Baseline characteristics of the patients were obtained including pregnancy related parameters such as gestational week (GW), birth mode, pregnancy-related complications and maternal comorbidities. ECMO-related variables recorded were indication for ECMO, the applied ECMO circuit, mode and configuration, days on ECMO, and survival of mother and child. Complications have been registered by clinical reports. Major bleeding was defined as a drop in hemoglobin of ≥2 g/dl/day or in transfusion of ≥2 packed red cells/24 h, or retroperitoneal, cerebral, or pulmonary bleeding.

### Statistical analysis

Continuous data are expressed as means with standard deviations (SD). Numbers are given as totals or in percentage as stated.

## Results

From the six participating ECMO centers, a total of 60 pregnancy or peripartal ECMO cases were identified making up for 1.55% of all ECMO cases at the participating centers. Mean patient age was 30.5 (±6.7) years. Indication for ECMO therapy was given in cases of severe respiratory and/or cardiac failure with need for extracorporeal support, in accordance to the ELSO (Extracorporeal life support organization) guidelines.^11^ The most common disease causing need for ECMO therapy during pregnancy was severe H1*N*1 influenza complicated with ARDS (*n* = 20). Further infectious complications including pneumonia other than H1*N*1 and sepsis required ECMO in 15 cases. Moreover, 6 (10%) patients had an indication for ECMO therapy due to worsening of an underlying disease, such as cystic fibrosis, pheochromocytoma, pre-existing mitral valve insufficiencies or structural heart defects. Pregnancy related disorders caused indication for ECMO in 14 patients, these indications are shown in Table 1.

**Table 1.** Indications for ECMO therapy, ECMO mode and outcome of mother and child.

ECMO-indication	Cases *n* = 60	VA-ECMO *n* = 14	VV-ECMO *n* = 46	Survival mother *n* = 52	Survival child *n* = 44
ARDS due to H1*N*1 influenza	20	0	20	18	11
Pneumonia other than H1*N*1 (bacterial/viral/aspiration)	8/1/1	0/0/0	8/1/1	8/1/1	8/0/1
Sepsis other than pneumonia	5	1	4	5	4
Eisenmenger-syndrome	2	0	2	2	1
Severe mitral valve insufficiency	2	2	0	1	1
Cystic fibrosis	1	0	1	1	1
Pheochromocytoma	1	1	0	1	1
Pulmonary embolism	5	3	2	4	4
Myocardial infarction	1	1	0	1	1
TRALI	2	0	2	2	2
Peripartum cardiomyopathy	4	3	1	3	4
Amniotic fluid embolism	2	1	1	2	2
HELLP-sydrom	2	0	2	1	2
Pre-eclampsia	1	0	1	1	1
Severe hemorrhage pregnancy/delivery associated	2	2	0	0	0

ECMO = Extracorporeal Membrane Oxygenation, ARDS = acute respiratory distress syndrome, VV = veno-venous, VA = veno-arterial, TRALI = Transfusion-related acute lung injury, HELLP = Haemolysis, Elevated Liver Enzyme Levels, Low Platelet Count.

The mode of ECMO support was VV ECMO for respiratory support in 46 cases (77%), while VA mode for circulatory support was applied less frequent (*n* = 14). Concerning ECMO configuration, the most common in VV ECMO was a drainage via the V. femoralis and a return cannula in V. jugularis (femoro-jugular) (*n* = 33). Alternative configurations for VV ECMO were femoro-femoral (*n* = 8), jugulo-femoral (*n* = 3), femoro-subclavian (*n* = 1), or a dual-lumen cannula via the jugular vein. The configuration for VA ECMO was a V. femoralis drainage cannula and an *A. femoralis* return (femoro-femoral) in all except one case, were a V. jugularis to *A. femoralis* configuration was applied.

Maternal survival of all reported peripartal ECMO cases was high (*n* = 52, 87%) and overall fetal survival was 73% (*n* = 44). For VA ECMO cases, maternal survival was 71% (10/14), for VV ECMO cases, 94% (43/46).

**Figure 2.**
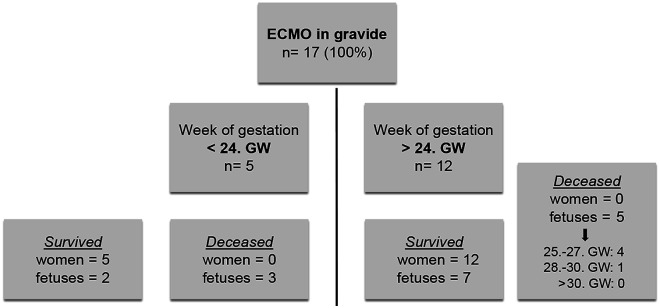
Outcome of women and children in cases of ECMO (extracorporeal membrane oxygenation) support during pregnancy in regards to week of gestation being <24 weeks or >24 weeks.

In regards of temporal connection to the birth, most women (*N* = 43) have been cannulated postpartum. All 14 VA ECMO patients were cannulated after giving birth. In VV ECMO, 65% were cannulated after delivery of the child. 17 patients were cannulated during pregnancy. Regarding fetal mortality eight fetuses/infants deceased before start of ECMO therapy. Reasons for fetal death included, acute hypoxemia of the mother before implementation of ECMO therapy with a secondary azidotic child, elective termination of pregnancy in severe maternal pre-existing illness or abortion in the first trimester before start of ECMO therapy. The fetal mortality in women who received ECMO therapy during pregnancy due to acute deterioration (respiratory/cardiac) was 47%. In the 25th-27th GW, four fetuses died, between the 28th-30th GW intrauterine fetal death occurred in one case under ongoing ECMO therapy. Above the 30th week of pregnancy, there was no more fetal mortality that could be associated with ECMO therapy. Outcome of women and fetuses of the in gravida ECMO cases are displayed in Figure 2.

Complications that occurred during ECMO therapy in our population included 14 major bleeding events and 14 thrombotic complications. Bleeding events included nine recorded abdominal bleedings. Thrombotic events included six deep vein thrombosis, one lung embolism, one arterial embolism in the leg and two ischemic colitis cases.

## Discussion

We present an international multicenter study with 60 ECMO patients in pregnancy and the peripartal period. Included in this study are six different centers from three different continents as shown in Figure 1. All participating centers are experienced ECMO centers and have dedicated ECMO programs since many years. Given the 10-years time span and six high volume ECMO centers our numbers show that pregnancy related cases are still rare, making up for about 1.5% of cases in the analyzed ECMO centers.

Our most important results are I) the diversity of indications for ECMO support during pregnancy, II) distribution of applied ECMO modes and configurations and III) maternal and fetal outcome.

The most frequent indication for need of extracorporeal support was ARDS with severe respiratory failure (60%), mostly secondary to influenza H1*N*1. With ARDS being the most frequent indication, thus the applied ECMO mode was VV in 77%. These numbers are in accordance with the published literature.^8,12,13^

In regards of circulatory support with VA ECMO peripartal cardiomyopathy and lung embolism were the most frequent indications.

The existing literature consist of a number of case reports, smaller case series and literature reviews on ECMO in pregnancy or the peripartal phase. The largest case series so far was reported by Lankford and colleagues^14^ who reported on 21 cases including six in gravida patients with good survival. A literature review on the topic by Moore et al. in 2016 identified a total of 45 cases, including 41 VV ECMO and four VA ECMO cases 8 and the most recent review by Ong et al., identified 97 cases from 90 publications 7. These numbers emphasize the lack of information on this specific group of patients and scenarios.

Our data shows that indications for ECMO in pregnancy and the peripartal period are various and decision for the ECMO mode to be applied decided based on the organ support needed. Indications including pneumonia and other infections leading to severe respiratory failure are well accepted indications for VV ECMO support independent of pregnancy 1.

Choice of ECMO mode in patients with thrombotic or amniotic fluid pulmonary embolism is depending on the circulatory and/or respiratory situation of the patient. If circulatory failure occurs VA ECMO is indicated, if severe hypoxemia is central VV ECMO can be chosen.^15^ Different scenarios of pulmonary embolism and ECMO support in the peripartal period are described in the literature.^16–18^ The benefit of VV ECMO in pulmonary embolism is sometimes discussed controversial as no circulatory support is provided. The pathophysiologic explanation is that hypoxemia increases the afterload of the right ventricle and if hypoxemia is treated through ECMO support the afterload is reduced - resulting in improved circulation. In our analysis five women with thrombotic embolism and two with amniotic fluid embolism were included. Mode of ECMO support was VV ECMO in four cases and VA ECMO in three cases.

In this study, four cases of peripartum cardiomyopathy, of whom three have received circulatory support (VA) and one respiratory support with VV ECMO, were included. This is in line with a recent case series by Djordjevic et al.,^19^ and a successful case report using VV ECMO in peripartal cardiomyopathy.^20^ Further we reported two cases of severe hemorrhage of which both patients died. However, in a retrospective case series in five women with severe hemorrhage from uterine atony, Huang and colleagues^21^ reported a good outcome in four of them (80%). Beside these reported peripartal cases, ECMO in acute hemorrhagic shock is a rare indication limited to case reports mostly in trauma patients,^22,23^ and this indication for ECMO may be regarded controversial. Therefore, no evidence exists, whether ECMO therapy has an indication in hemorrhagic shock, in pregnancy or in general. The decision to start ECMO therapy in such emergency scenarios should be made jointly by the team of obstetrician, anesthesiologist and ECMO specialist. Benefit of ECMO support in hemorrhagic shock patients might be secondary as mass-transfusion and volume substitution are the mainstay of therapy. However ECMO support will be necessary if circulatory failure persists or an ARDS develops following mass-transfusion.

ECMO configurations are possible in various ways, with femoral-jugular being the most used in VV and femoro-femoral in VA ECMO. The choice of cannulation sites should be made by the ECMO team according to the need of the patient and associated risks.

Our reported survival of mothers of 87% is high and in line with reported survival rates of 77.2% in previous observantional studies and 90.7% in all reported cases as shown in a systematic review by Zhang et al.^7,24^ In VA ECMO cases survival is lower with 71%, however still outcome in peripartal cases are good compared to about 50% survival in adult ECMO at large.^25–27^ Nevertheless, special focus should be on cases with fatal outcome. Maybe some deaths could be prevented by improved decision-making. Children <24 GW are not likely to survive outside the uterus. The goal should be to prolong pregnancy and intense monitoring of the unborn should be applied. Thoughtful decisions should be made regarding children of ^25–29^ GW, as these children in general have good chances of survival (84%).^28^ In our data in five cases of pregnancies with >24 GW intrauterine fetal demise occurred during ECMO therapy. These are cases in which it should be discussed if a cesarean delivery might have been possible before initiation of ECMO support. However, sometimes impairment of the fetus occurs before implementation of ECMO therapy due to the critical state (e.g hypoxemia) of the mother, therefore state fetus has to be checked on as soon as possible by an obstetrician.

ECMO support in general is associated with an increased risk for bleeding and thrombotic events^29,30^ and these complications have a critical impact on outcome. Although not systematically recorded ECMO associated complications were high in our population, which is in contrast to our good outcome. This might be explained by in general higher bleeding risks in the peripartal period and the overall better condition of young women compared to other adult ECMO populations. Outcome in young women of reproductive age is reported to be even better independent of pregnancy.^31^

Although our data gives a good estimate of survival rates in peripartal ECMO cases in experienced centers, our study has some limitations: The retrospective nature of data acquisition does not give an actual incidence of peripartal cases. Complications and outcome have not been recorded systematically. Detailed information on fetal death is missing. Numbers in need for ECMO support may be underestimated as the participating ECMO centers are not directly connected with surrounding obstetric units, therefore some cases might have not received ECMO treatment due to unavailability or missing networks. Variability of mobile ECMO services, networking and availability of ECMO resources for obstetricians vary a lot.^32^ Data are not generalizable, as only experienced ECMO centers participated in the study and ECMO indications are heterogenous. Of note is further that 18 patients from the center in New York, USA, have been published previously.^33^

In conclusion, ECMO can be considered as a rescue therapy in pregnancy and the peripartal period as it has shown to be feasible. Indications in this population are diverse and, apart from hemorrhagic shock, survival is high. The study population are young women with a high life expectancy and all survivors left the hospital in good health.

It is important to know that ECMO can be a life saving treatment for mother and child. Therefore it is crucial that in case of an obstetric emergency some circumstances have to be full-filled: 1. The attending clinician has to be aware of the possibility of ECMO support in any given situation; 2. ECMO support must be requested; 3. The ECMO resource must be available and ECMO commenced in a timely manner.

Initiation of ECMO support as well as further patient care in peripartel cases should be a team effort by obstetricians, anesthesiologists and ECMO specialists. Especially in ongoing pregnancy continually discussion on the best management of mother and child is decisive.
